# Bis(1-carbamimidoyl-2-ethyl­isourea)copper(II) dinitrate

**DOI:** 10.1107/S1600536809041932

**Published:** 2009-10-17

**Authors:** Atittaya Meenongwa, Unchulee Chaveerach, Chaveng Pakawatchai

**Affiliations:** a Department of Chemistry and Center of Excellence for Innovation in Chemistry, Faculty of Science, Khon Kaen University, Khon Kaen 40002, Thailand; b Department of Chemistry, Prince of Songkla University, Hatyai 90112, Thailand

## Abstract

The copper(II) complex, [Cu(C_4_H_10_N_4_O)_2_](NO_3_)_2_ or [Cu(*L* ^1*e*^)_2_](NO_3_)_2_, where *L* ^1*e*^ is 1-carbamimidoyl-2-ethyl­isourea, was obtained from a 1:2 molar ratio of copper(II) nitrate hemipenta­hydrate with 2-cyano­guanidine in ethanol. The crystal structure consists of the centrosymmetric [Cu(*L* ^1*e*^)_2_]^2+^ cation and two NO_3_^−^ counter-anions. The cation exhibits four-coordinate bonding of the two *N*,*N*-bidentate ligands and the Cu^II^ atom through the N-donor atoms, yielding a square-planar CuN_4_ geometry. Inter­molecular N—H⋯O hydrogen bonds link between the cation and and counter-anion, forming a two-dimentional layered structure extending parallel to (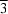
01).

## Related literature

For a copper(II) complex containg the same *N,N*-bidentate 1-carbamimidoyl-2-ethyl­isourea ligand but with the charge balance provided by two chloride and perchlorate anions, see: Begley *et al.* (1986 ▶); Meenongwa *et al.* (2009 ▶).
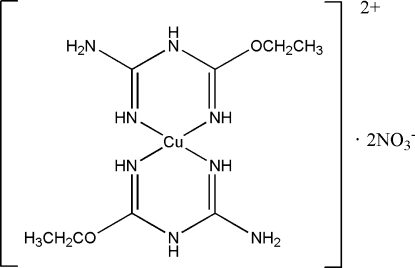


## Experimental

### 

#### Crystal data

[Cu(C_4_H_10_N_4_O)_2_](NO_3_)_2_
*M* *_r_* = 447.89
Monoclinic, 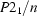

*a* = 5.2547 (6) Å
*b* = 14.0087 (15) Å
*c* = 12.1511 (13) Å
β = 96.982 (2)°
*V* = 887.83 (17) Å^3^
*Z* = 2
Mo *K*α radiation
μ = 1.29 mm^−1^
*T* = 293 K
0.26 × 0.16 × 0.11 mm

#### Data collection

Bruker SMART APEX CCD area detector diffractometer
Absorption correction: multi-scan (*SADABS*; Bruker, 2003 ▶) *T*_min_ = 0.793, *T*_max_ = 1.00
11998 measured reflections
2206 independent reflections
1810 reflections with *I* > 2σ(*I*)
*R*_int_ = 0.028

#### Refinement

*R*[*F* ^2^ > 2σ(*F* ^2^)] = 0.035
*wR*(*F* ^2^) = 0.102
*S* = 1.05
2206 reflections
125 parameters
3 restraints
H-atom parameters constrained
Δρ_max_ = 0.73 e Å^−3^
Δρ_min_ = −0.27 e Å^−3^

### 

Data collection: *SMART* (Bruker, 1998 ▶); cell refinement: *SAINT* (Bruker, 2003 ▶); data reduction: *SAINT*; program(s) used to solve structure: *SHELXS97* (Sheldrick, 2008 ▶); program(s) used to refine structure: *SHELXL97* (Sheldrick, 2008 ▶); molecular graphics: *SHELXTL* (Sheldrick, 2008 ▶); software used to prepare material for publication: *enCIFer* (Allen *et al.*, 2004 ▶) and *publCIF* (Westrip, 2009 ▶).

## Supplementary Material

Crystal structure: contains datablocks global, I. DOI: 10.1107/S1600536809041932/ds2008sup1.cif

Structure factors: contains datablocks I. DOI: 10.1107/S1600536809041932/ds2008Isup2.hkl

Additional supplementary materials:  crystallographic information; 3D view; checkCIF report

## Figures and Tables

**Table:** 

Cu1—N1	1.932 (4)
Cu1—N1^i^	1.932 (4)
Cu1—N2	1.967 (4)
Cu1—N2^i^	1.967 (4)

**Table:** 

N1—Cu1—N1^i^	180.0
N1—Cu1—N2	88.33 (19)
N1^i^—Cu1—N2	91.67 (19)
N1—Cu1—N2^i^	91.67 (19)
N1^i^—Cu1—N2^i^	88.33 (19)
N2—Cu1—N2^i^	179.999 (1)

Symmetry code: (i) 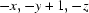.

**Table 2:** Hydrogen-bond geometry (Å, °)

*D*—H⋯*A*	*D*—H	H⋯*A*	*D*⋯*A*	*D*—H⋯*A*
N1—H1⋯O2^ii^	0.86	2.05	2.904 (7)	170
N2—H2⋯O2^iii^	0.86	2.18	3.009 (6)	163
N3—H3⋯O4	0.86	2.14	2.979 (6)	165
N4—H45⋯O3	0.86	2.06	2.917 (7)	171

Symmetry codes: (ii) 
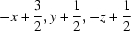
; (iii) 
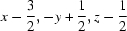
.

**Fig. 1.**
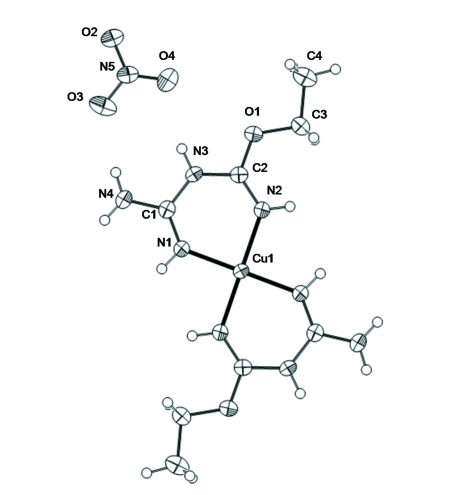
View of the title copper(II) complex, showing the atom numbering of the cationic [Cu(L1e)2]2+ moiety of [Cu(L1e)2](NO3)2. Displacement ellipsoids are drawn at the 50% probability level.

**Fig. 2.**
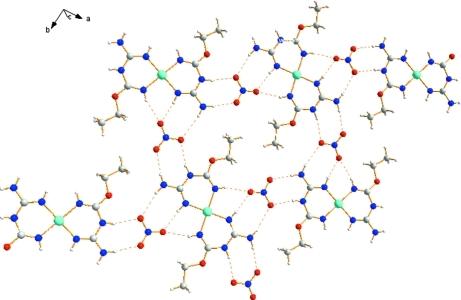
The crystal structure of [Cu(L1e)2](NO3)2 showing the linking of [Cu(L1e)2]2+ cation and NO3- counteranion along b axis. Hydrogen bonds are presented as a dashed lines.

## supplementary crystallographic information

### Comment

Herein, we report the structure of [Cu(*L*^1^*^e^*)_2_](NO_3_)_2_, which was obtained from the similar procedure as previously reported by Meenongwa *et al.* (2009), but using copper(II) nitrate hemipentahydrate. Structural determination on the title complex reveals a centrosymmetric [Cu(*L*^1^*^e^*)_2_]^2+^ cation and two NO_3_^-^ counteranions. Fig. 1 shows the [Cu(*L*^1^*^e^*)_2_]^2+^ moiety. The square-planar CuN_4_ geometry is yielded by the coordination of the two *N,N*-bidentate ligands (Table 1) with Cu— N bond distances of 1.9313 (16) - 1.9650 (17) Å. Moreover, NO_3_^-^ anions also contact to the neighboring cationic units by various hydrogen bonds of the type N—H···O (nitrate) to give a two dimentional layered structure (Fig. 2) as observed in the previuos [Cu(*L*^1^*^e^*)_2_](ClO_4_)_2_ complex.

### Experimental

The initial product of the title complex was obtained from the reaction of 2-cyanoguanidine (0.1682 g, 2 mmol, Aldrich, 99%) with copper(II) nitrate hemipentahydrate (0.2325 g, 1 mmol, Sigma-Aldrich, 98%). The reaction was carried out in ethanol under refluxing condition for 24 h. The reddish-pink precipitate thus formed was isolated by filtration. The red block shaped single crystals were grown by slow vapor phase diffusion of methanol-ethanol solution of this products into toluene medium at room temperature for a week.

### Refinement

The crystal structure refinement was initially performed by direct method to locate the structural model. All non-hydrogen atoms were refined anisotropically. All hydrogen atoms were positioned geometrically and refined as riding atoms, with N—H = 0.86, *C*—*H*(methyl) = 0.96 and *C*—*H*(methylene) = 0.97 Å, and approximation with *U*_iso_(H) = *xU*_eq_(C, N), where *x* = 1.5 for methyl H atoms and 1.2 for all others.

### Crystal data

**Table:**

[Cu(C_4_H_10_N_4_O)_2_](NO_3_)_2_	*F*(000) = 462
*M**_r_* = 447.89	*D*_x_ = 1.675 Mg m^−^^3^
Monoclinic, *P*2_1_/*n*	Mo *K*α radiation, λ = 0.71073 Å
Hall symbol: -P 2yn	Cell parameters from 4098 reflections
*a* = 5.2547 (6) Å	θ = 2.9–27.1°
*b* = 14.0087 (15) Å	µ = 1.29 mm^−^^1^
*c* = 12.1511 (13) Å	*T* = 293 K
β = 96.982 (2)°	Block, red
*V* = 887.83 (17) Å^3^	0.26 × 0.16 × 0.11 mm
*Z* = 2	

### Data collection

**Table:**

Bruker SMART APEX CCD area detector diffractometer	2206 independent reflections
Radiation source: fine-focus sealed tube	1810 reflections with *I* > 2σ(*I*)
graphite	*R*_int_ = 0.028
Frames each covering 0.3 ° in ω scans	θ_max_ = 28.3°, θ_min_ = 2.2°
Absorption correction: multi-scan (*SADABS*; Bruker, 2003)	*h* = −7→7
*T*_min_ = 0.793, *T*_max_ = 1.00	*k* = −18→18
11998 measured reflections	*l* = −16→16

### Refinement

**Table:**

Refinement on *F*^2^	Primary atom site location: structure-invariant direct methods
Least-squares matrix: full	Secondary atom site location: difference Fourier map
*R*[*F*^2^ > 2σ(*F*^2^)] = 0.035	Hydrogen site location: inferred from neighbouring sites
*wR*(*F*^2^) = 0.102	H-atom parameters constrained
*S* = 1.05	*w* = 1/[σ^2^(*F*_o_^2^) + (0.0621*P*)^2^ + 0.2477*P*] where *P* = (*F*_o_^2^ + 2*F*_c_^2^)/3
2206 reflections	(Δ/σ)_max_ < 0.001
125 parameters	Δρ_max_ = 0.73 e Å^−^^3^
3 restraints	Δρ_min_ = −0.27 e Å^−^^3^

### Special details

**Table:**

Geometry. All e.s.d.'s (except the e.s.d. in the dihedral angle between two l.s. planes) are estimated using the full covariance matrix. The cell e.s.d.'s are taken into account individually in the estimation of e.s.d.'s in distances, angles and torsion angles; correlations between e.s.d.'s in cell parameters are only used when they are defined by crystal symmetry. An approximate (isotropic) treatment of cell e.s.d.'s is used for estimating e.s.d.'s involving l.s. planes.
Refinement. Refinement of *F*^2^ against ALL reflections. The weighted *R*-factor *wR* and goodness of fit *S* are based on *F*^2^, conventional *R*-factors *R* are based on *F*, with *F* set to zero for negative *F*^2^. The threshold expression of *F*^2^ > σ(*F*^2^) is used only for calculating *R*-factors(gt) *etc*. and is not relevant to the choice of reflections for refinement. *R*-factors based on *F*^2^ are statistically about twice as large as those based on *F*, and *R*- factors based on ALL data will be even larger.

### Fractional atomic coordinates and isotropic or equivalent isotropic displacement parameters (Å^2^)

**Table:**

	*x*	*y*	*z*	*U*_iso_*/*U*_eq_	
Cu1	0.0000	0.5000	0.0000	0.0348 (3)	
N1	0.3075 (8)	0.5323 (3)	0.0966 (4)	0.0406 (10)	
H1	0.3311	0.5925	0.1070	0.049*	
N2	0.0819 (9)	0.3645 (3)	0.0282 (4)	0.0434 (11)	
H2	−0.0240	0.3241	−0.0050	0.052*	
N3	0.4651 (8)	0.3829 (3)	0.1454 (4)	0.0408 (10)	
H3	0.5879	0.3520	0.1829	0.049*	
O1	0.3213 (8)	0.2382 (3)	0.1101 (4)	0.0516 (11)	
N4	0.6968 (10)	0.5138 (3)	0.2062 (5)	0.0541 (14)	
H44	0.7222	0.5744	0.2107	0.065*	
H45	0.8084	0.4751	0.2391	0.065*	
C1	0.4823 (10)	0.4794 (4)	0.1477 (5)	0.0378 (11)	
C3	0.1450 (12)	0.1670 (3)	0.0590 (5)	0.0506 (14)	
H31	0.1250	0.1730	−0.0211	0.061*	
H32	−0.0219	0.1733	0.0847	0.061*	
C2	0.2729 (9)	0.3293 (4)	0.0898 (4)	0.0383 (11)	
N5	0.9978 (9)	0.2854 (3)	0.3317 (4)	0.0479 (11)	
O2	1.1439 (11)	0.2388 (4)	0.3966 (5)	0.090 (2)	
C4	0.2662 (17)	0.0734 (4)	0.0945 (7)	0.075 (2)	
H41	0.4291	0.0681	0.0668	0.113*	
H43	0.1567	0.0222	0.0654	0.113*	
H42	0.2904	0.0700	0.1740	0.113*	
O4	0.8192 (10)	0.2461 (4)	0.2742 (4)	0.0705 (14)	
O3	1.0295 (12)	0.3715 (3)	0.3254 (5)	0.0863 (18)	

### Atomic displacement parameters (Å^2^)

**Table:**

	*U*^11^	*U*^22^	*U*^33^	*U*^12^	*U*^13^	*U*^23^
Cu1	0.0290 (5)	0.0239 (5)	0.0475 (6)	0.0021 (3)	−0.0123 (3)	0.0011 (3)
N1	0.034 (2)	0.0256 (19)	0.057 (3)	0.0008 (17)	−0.0160 (19)	−0.0010 (18)
N2	0.038 (2)	0.0252 (19)	0.061 (3)	−0.0003 (17)	−0.0196 (19)	−0.0003 (18)
N3	0.032 (2)	0.029 (2)	0.055 (3)	0.0029 (16)	−0.0159 (18)	0.0043 (18)
O1	0.048 (2)	0.0249 (17)	0.075 (3)	0.0016 (15)	−0.0226 (19)	0.0038 (17)
N4	0.041 (3)	0.035 (2)	0.077 (4)	−0.0022 (18)	−0.029 (3)	0.002 (2)
C1	0.032 (2)	0.031 (2)	0.047 (3)	−0.0008 (18)	−0.008 (2)	−0.0002 (19)
C3	0.053 (3)	0.027 (2)	0.066 (4)	−0.001 (2)	−0.017 (3)	−0.001 (2)
C2	0.035 (2)	0.027 (2)	0.050 (3)	0.0019 (18)	−0.008 (2)	0.0025 (19)
N5	0.049 (3)	0.035 (2)	0.056 (3)	0.0082 (19)	−0.011 (2)	0.005 (2)
O2	0.090 (4)	0.043 (3)	0.119 (5)	0.006 (3)	−0.064 (3)	0.012 (3)
C4	0.084 (5)	0.031 (3)	0.103 (6)	0.004 (3)	−0.026 (4)	0.002 (3)
O4	0.057 (3)	0.059 (3)	0.086 (3)	−0.001 (2)	−0.029 (2)	0.000 (2)
O3	0.102 (4)	0.032 (2)	0.117 (5)	0.004 (2)	−0.023 (3)	0.015 (3)

### Geometric parameters (Å, °)

**Table:**

Cu1—N1	1.932 (4)	N4—C1	1.347 (7)
Cu1—N1^i^	1.932 (4)	N4—H44	0.8600
Cu1—N2	1.967 (4)	N4—H45	0.8600
Cu1—N2^i^	1.967 (4)	C3—C4	1.498 (8)
N1—C1	1.281 (7)	C3—H31	0.9700
N1—H1	0.8600	C3—H32	0.9700
N2—C2	1.276 (6)	N5—O3	1.221 (6)
N2—H2	0.8600	N5—O2	1.221 (6)
N3—C1	1.355 (7)	N5—O4	1.230 (6)
N3—C2	1.369 (6)	C4—H41	0.9600
N3—H3	0.8600	C4—H43	0.9600
O1—C2	1.319 (6)	C4—H42	0.9600
O1—C3	1.449 (6)		
			
N1—Cu1—N1^i^	180.0	N1—C1—N3	121.7 (5)
N1—Cu1—N2	88.33 (19)	N4—C1—N3	114.7 (5)
N1^i^—Cu1—N2	91.67 (19)	O1—C3—C4	104.6 (5)
N1—Cu1—N2^i^	91.67 (19)	O1—C3—H31	110.8
N1^i^—Cu1—N2^i^	88.33 (19)	C4—C3—H31	110.8
N2—Cu1—N2^i^	179.999 (1)	O1—C3—H32	110.8
C1—N1—Cu1	131.1 (4)	C4—C3—H32	110.8
C1—N1—H1	114.5	H31—C3—H32	108.9
Cu1—N1—H1	114.5	N2—C2—O1	127.1 (5)
C2—N2—Cu1	128.0 (4)	N2—C2—N3	123.9 (4)
C2—N2—H2	116.0	O1—C2—N3	108.9 (4)
Cu1—N2—H2	116.0	O3—N5—O2	119.3 (6)
C1—N3—C2	126.9 (4)	O3—N5—O4	120.5 (5)
C1—N3—H3	116.6	O2—N5—O4	120.3 (5)
C2—N3—H3	116.6	C3—C4—H41	109.5
C2—O1—C3	119.2 (4)	C3—C4—H43	109.5
C1—N4—H44	120.0	H41—C4—H43	109.5
C1—N4—H45	120.0	C3—C4—H42	109.5
H44—N4—H45	120.0	H41—C4—H42	109.5
N1—C1—N4	123.7 (5)	H43—C4—H42	109.5
			
N2—Cu1—N1—C1	3.1 (6)	C2—O1—C3—C4	176.9 (6)
N2^i^—Cu1—N1—C1	−176.9 (6)	Cu1—N2—C2—O1	178.1 (4)
N1—Cu1—N2—C2	0.4 (5)	Cu1—N2—C2—N3	−2.6 (9)
N1^i^—Cu1—N2—C2	−179.6 (5)	C3—O1—C2—N2	0.1 (9)
Cu1—N1—C1—N4	174.9 (5)	C3—O1—C2—N3	−179.3 (5)
Cu1—N1—C1—N3	−4.2 (9)	C1—N3—C2—N2	2.1 (10)
C2—N3—C1—N1	1.3 (10)	C1—N3—C2—O1	−178.5 (5)
C2—N3—C1—N4	−177.9 (6)		

Symmetry codes: (i) −*x*, −*y*+1, −*z*.

### Hydrogen-bond geometry (Å, °)

**Table:**

*D*—H···*A*	*D*—H	H···*A*	*D*···*A*	*D*—H···*A*
N1—H1···O2^ii^	0.86	2.05	2.904 (7)	170
N2—H2···O2^iii^	0.86	2.18	3.009 (6)	163
N3—H3···O4	0.86	2.14	2.979 (6)	165
N4—H45···O3	0.86	2.06	2.917 (7)	171

Symmetry codes: (ii) −*x*+3/2, *y*+1/2, −*z*+1/2; (iii) *x*−3/2, −*y*+1/2, *z*−1/2.
